# Biosynthesis of Magnetite Nanoparticles Mediated by Chia Mucilage and Its Co-Encapsulation with *Lactobacillus rhamnosus* GG by Spray Drying: Evaluation Under Simulated Gastrointestinal Digestion

**DOI:** 10.3390/foods15081304

**Published:** 2026-04-09

**Authors:** Victor Bascur, Carolina Shene, Olga Rubilar, Mariela Bustamante

**Affiliations:** 1 Doctorate in Sciences Engineering with Specialization in Bioprocesses, Universidad de La Frontera, Temuco 4811230, Chile; v.bascur01@ufromail.cl; 2 Department of Chemical Engineering, Universidad de La Frontera, Av. Francisco Salazar 01145, Temuco 4811230, Chile; carolina.shene@ufrontera.cl (C.S.); olga.rubilar@ufrontera.cl (O.R.); 3 Center of Food Biotechnology and Bioseparations, BIOREN, and Centre of Biotechnology and Bioengineering (CeBiB), Universidad de La Frontera, Temuco 4811230, Chile; 4 Centro de Excelencia en Investigación Biotecnológica Aplicada al Medio Ambiente (CIBAMA), Universidad de La Frontera, Temuco 4811230, Chile

**Keywords:** Fe_3_O_4_, chia seed mucilage, *Lactobacillus rhamnosus* GG, spray drying, gastrointestinal digestion

## Abstract

This study investigated the biosynthesis of magnetite nanoparticles mediated by chia mucilage (CM-Fe_3_O_4_ NPs) and their application in the co-encapsulation of *Lactobacillus rhamnosus* GG (LGG) using spray drying. CM-Fe_3_O_4_ NPs were synthesized by combining CM extract with iron salts, in which hydroxyl and carbonyl groups of CM acted as natural ligands for Fe^2+^/Fe^3+^ ions. A response surface design was applied to optimize synthesis parameters, focusing on size distribution and zeta potential, and confirming the influence of pH on colloidal stability. Characterization by FE-SEM, DLS, XRD, UV-Vis, and FTIR revealed spherical particles with an inorganic core (50–300 nm) and a hydrated organic coating (600–900 nm), consistent with a spinel structure functionalized by CM. Spray-drying encapsulation tests showed that incorporating CM-Fe_3_O_4_ NPs did not compromise bacterial viability, maintaining optimal moisture content and survival. Growth curves and confocal microscopy corroborated the physiological compatibility of the nanoparticles, with no alterations in LGG morphology or growth dynamics. Under simulated gastrointestinal conditions, co-encapsulated microcapsules exhibited slightly improved survival in the gastric phase and significantly greater viability in the initial intestinal phase. These results suggest that CM-Fe_3_O_4_ NPs modulate matrix degradation and promote controlled release, ensuring therapeutic concentrations of LGG in the intestine. Overall, the CM-Fe_3_O_4_ nanocomposite system integrates the protective properties of biopolymers with the functional advantages of iron nanoparticles, offering dual functionality: probiotic stabilization and potential iron supplementation. This innovative, food-grade approach supports the development of next-generation functional foods with combined therapeutic and nutritional benefits.

## 1. Introduction

Probiotic bacteria from the genera *Lactobacillus* and *Bifidobacterium* are widely used in the food industry for their well-established health-promoting benefits. These microorganisms help with gastrointestinal regulation [1], modulation of the intestinal microbiota [1], and stimulation of the immune system [2], among other benefits. Their inclusion in functional foods has become a key strategy for enhancing consumer health and well-being. Among the many probiotic strains, *Lactobacillus rhamnosus* GG (LGG) is one of the most extensively studied. Research has focused on its stability under storage conditions [3], its resistance to thermal processing [4,5], and its survival under simulated gastrointestinal environments [4,5]. For LGG to exert therapeutic effects, probiotic products must deliver between 10^6^ and 10^7^ CFU/g at the time of consumption [6]. Achieving this concentration requires bacteria to survive gastrointestinal transit and overcome adverse environmental or technological stresses, which remains a major challenge for industrial applications [7].

To address these limitations, encapsulation technologies have been developed to protect probiotics and improve their survivability. Encapsulation offers a physical barrier against environmental stressors, enhancing microbial stability during processing, storage, and digestion [7,8,9]. Encapsulation strategies using polymers like chitosan and alginate are widely reported for nanoparticle synthesis and probiotic delivery, offering useful benchmarks for evaluating alternative biopolymers [10,11]. Additionally, pectin-based systems have been studied as food-grade encapsulation matrices, reinforcing the broader importance of natural polysaccharides in probiotic stabilization [12,13]. Among the available techniques, spray drying (SD) has become one of the most effective and scalable methods for probiotic encapsulation, providing benefits in terms of cost, reproducibility, and industrial feasibility [8]. Bustamante et al. [3] reported that encapsulation of probiotic bacteria in cross-linked alginate matrices (CLAMs) supplemented with chia seed mucilage (CM) resulted in survival rates above 92% after processing and sustained viability of 9 log CFU/g after 90 days of storage at 4 °C. Similarly, Bascur et al. [5] demonstrated that LGG encapsulated in CLAMs supplemented with CM preserved during gastrointestinal digestion (above 6 log CFU/g) and to confer thermal stability under heat stress conditions at 80 °C for 5 min. These findings highlight the potential of combining alginate matrices with natural biopolymers, such as CM, to enhance probiotic resilience under industrially relevant conditions.

Despite these advances, the gastric phase still poses a significant barrier to probiotic survival. The stomach’s highly acidic environment, marked by the presence of hydrogen ions, damages cell membranes and causes cell death [14]. This challenge has spurred the investigation of new protective agents that improve probiotic resistance to gastric conditions’ acidity. In this context, iron oxide nanoparticles (IONs) have shown promising results. Novin et al. [15] demonstrated that magnetic immobilization of *Lactobacillus acidophilus* with IONs increased bacterial viability at low pH, with a 1.8 log increase when 360 µg/mL of IONs were added to the culture medium. Likewise, Ghibaudo et al. [16] reported that probiotic bacteria encapsulated in pectin supplemented with IONs maintained high viability in the gastric phase (11.33 ± 0.08 log CFU/mL), with no significant differences compared to the oral phase. These studies suggest that IONs can act as protective agents, enhancing probiotic survival in acidic environments and potentially expanding their applications in functional foods and pharmaceuticals.

However, when adding IONs to food or pharmaceutical products, it is crucial to minimize potential cytotoxic effects [17]. Conventional synthesis methods often use chemical precursors that may pose risks to human health [18]. To address these issues, green synthesis methods using plant-derived compounds have been suggested as safer and more sustainable options [19,20]. Plant-based biopolymers not only decrease toxicity but also offer biocompatibility and functionality. In this context, CM is a biopolymer of importance because of its food-grade biocompatibility [21,22], gelling properties [23], and ability to interact with nanoparticles [24]. CM-Fe_3_O_4_ refers to magnetite nanoparticles synthesized using CM, where hydroxyl and carbonyl groups serve as natural ligands for Fe^2+^/Fe^3+^ ions, enabling an environmentally friendly and food-grade synthesis route. CM also acts as an intermediate stabilizer due to its water-binding capacity and ability to retain microorganisms within a hydrated matrix. Chitosan-modified Fe_3_O_4_ has been reported to improve coordination with biological structures [25]; in our study, CM offers a food-grade alternative with similar stabilizing effects. Microbial encapsulation plays a critical role in enhancing biological efficacy, protecting against adverse gastrointestinal conditions, and ensuring therapeutic concentrations of probiotics in the intestine. To validate this approach, encapsulation and performance tests were conducted under simulated gastrointestinal digestion conditions to evaluate both nanoparticle stability and LGG survival. While promising, challenges such as variability in mucilage composition and long-term storage stability remain, highlighting the need for further optimization.

Based on this background, the objective of the present study was to biosynthesize magnetite-type IONs (CM-Fe_3_O_4_) mediated by chia mucilage and then co-encapsulate them with LGG using spray drying. The viability of LGG was evaluated under simulated gastrointestinal digestion to assess the protective effect of CM-Fe_3_O_4_ nanoparticles within the encapsulation matrices. This approach combines the benefits of biopolymer-based encapsulation with the protective properties of IONs to enhance probiotic survival in challenging gastrointestinal conditions and support the development of next-generation functional foods. Therefore, this study addresses a research gap by combining mucilage-mediated nanoparticle synthesis with probiotic encapsulation under physiologically relevant conditions, contributing to the development of innovative functional food systems that stabilize probiotics and offer potential iron supplementation.

## 2. Materials and Methods

### 2.1. Materials

*Lactobacillus rhamnosus* GG ATCC 53103 (LGG) was obtained from the American Type Culture Collection (Rockville, MD, USA). Chia seeds (*Salvia hispanica* L.) were purchased from Temuco Alimentos, Temuco, Chile. MRS broth (BD, Baltimore, MD, USA), sodium alginate (Sigma-Aldrich, St. Louis, MO, USA), succinic acid (Merck, Darmstadt, Germany), NH_4_OH (Sigma-Aldrich, St. Louis, MO, USA), CaHPO_4_ (Sigma-Aldrich, St. Louis, MO, USA), FeCl_3_ (Sigma-Aldrich, St. Louis, MO, USA), and FeCl_2_ (Sigma-Aldrich, St. Louis, MO, USA) were used in all experiments.

### 2.2. Synthesis of CM-Fe_3_O_4_ NPs

#### 2.2.1. CM Extraction

CM was extracted following Bustamante et al. [26] with some modifications. Seeds were treated with hot distilled water at 80 °C and pH 6.0 for 2 h, at a 1:40 (*w*/*v*) ratio. The mucilage was dried, milled, sieved (0.425 mm mesh), and stored at −20 °C until use.

#### 2.2.2. Effect of Iron Salts Concentration and pH on the Biosynthesis of CM-Fe_3_O_4_ NPs

The effects of pH and FeCl3 and FeCl2 concentrations on nanoparticle synthesis, specifically the size distribution and zeta potential, were evaluated. For this purpose, a 1 mL aliquot of CM extract was taken and added to a 100 mL Erlenmeyer flask. Then, the iron salts (FeCl_3_ and FeCl_2_) were added in molar proportions (40/20; 60/30; 80/40), and the pH was adjusted (9, 10, and 11), according to each treatment of the experimental design. Finally, they were incubated in an orbital shaker at 150 rpm for 2 h. They were later dried (60 °C for 12 h), and finally, the powder was extracted and stored at room temperature for later analysis.

An experimental design based on Response Surface Methodology (RSM) was applied to optimize the synthesis conditions of the nanoparticles and to evaluate the size distribution and zeta potential of the CM-Fe_3_O_4_ NPs. Two independent variables were considered in the experimental design: iron salt concentration (2:1 mM) and pH (9–11). The optimal concentrations of these two components were determined using a central composite face-centered design. The variables were considered at three working levels for each, with three replicates at the central point, i.e., to construct response surface models, a set of 11 experiments was selected. Design-Expert v.6.0 software was used to fit the experimental data to polynomial equations. Each treatment was performed with three biological replicates, and each replicate was analysed in triplicate technical measurements for particle size and zeta potential.

### 2.3. Characterization of CM-Fe_3_O_4_ NPs

#### 2.3.1. Dynamic Light Scattering and Zeta Potential

The hydrodynamic diameters of the scatterings of the CM-Fe_3_O_4_ NPs were measured using Zetasizer Nano ZS90 System (Malvern Instruments, Malvern, UK). Samples were diluted in distilled water (1 mg/mL). Data are presented as an average of three measurements. Averaged sizes were obtained by cumulative analysis of autocorrelation functions. Measurements of hydrodynamic diameter and zeta potential were conducted in three biological replicates, with three technical replicates per sample.

#### 2.3.2. Morphology and Size of CM-Fe_3_O_4_ NPs

Morphology was observed using a GeminiSEM 360 (ZEISS, Oberkochen, Germany), a field-emission scanning electron microscope (FE-SEM). The images were acquired at an acceleration voltage of 5.00 kV, a working distance of 8.9 mm, and with the InLens detector, which is suitable for high surface resolution. The magnification used was 30,000×, with a capture area of 3841 µm × 2858 µm and a reference scale of 400 nm.

#### 2.3.3. Spectra Analysis of CM-Fe_3_O_4_ NPs

The crystal structure of nanoparticles was determined by X-ray diffraction (XRD) (Siemens D5000, Munich, Germany) at 2θ between 10° and 90°.

The adsorption of surface plasmon CM-Fe_3_O_4_ NPs was confirmed by UV-visible adsorption spectroscopy, performed on a spectrophotometer (Genesys 10S, Thermo Scientific, Waltham, MA, USA). to identify characteristic adsorption peaks indicative of nanoparticle formation. A stable colloidal suspension was prepared by dispersing 20 mg of CM-Fe_3_O_4_ NPs powder in 5 mL of deionized water. The suspension was then sonicated for 10 min to ensure uniform dispersion. Then, suspension (1 mL) was placed in a cuvette and inserted into the spectrophotometer for analysis over the wavelength range of 200 to 700 nm.

The determination of the functional groups present in the structure was carried out using Fourier transform infrared spectroscopy (FT-IR) with a Bruker Vector 22 (Bruker Optics GmbH, Inc., Ettlingen, Germany) spectrometer, operating within a frequency range of 4000–250 cm^−1^, and utilizing KBr pellets as the matrix. The samples were analysed using transmittance mode to detect the presence of specific functional groups. Representative spectra and micrographs were obtained from three independent biological replicates.

### 2.4. Preparation of LGG with CM-Fe_3_O_4_ NPs

LGG was pre-cultured twice with a 5% (*v*/*v*) inoculum in MRS broth (5 mL) and incubated at 37 °C for 12 h. LGG was grown in 200 mL MRS broth (37 °C, 14 h). A total of 18 mg of CM-Fe_3_O_4_ NPs was dispersed in sterile distilled water and subjected to ultrasound at 20 kHz for 2 min. Adhesion of nanoparticles to LGG cells was performed as described by Novin et al. [15]. Suspensions were combined with LGG in MRS medium and incubated on a shaker at 200 rpm and 37 °C. After immobilization, the mixture was adjusted to a final volume of 50 mL with MRS medium, corresponding to a concentration of 360 µg/mL of CM-Fe_3_O_4_ NPs. The nanoparticle concentration (360 µg/mL) was selected based on preliminary optimization and literature reports. Cells in late log phase were harvested by centrifugation (4100 rpm, 4 °C, and 15 min). Biomass was washed with sterile distilled water and centrifuged again as described above. Finally, the LGG were re-suspended in sterile distilled water and added to the encapsulating solution. The adhesion of the CM-Fe_3_O_4_ NPs was confirmed by FE-SEM as described in Section 2.3.2. The adhesion assays were carried out in three biological replicates, and each replicate was confirmed by FE-SEM with two technical replicates.

### 2.5. Preparation of the Encapsulating Solution of M-LGG and M-LGG-CM-Fe_3_O_4_

The encapsulation solution, M-LGG-CM-Fe_3_O_4_, was prepared in 120 mL of distilled water according to Bascur et al. [5]; 100 mL were prepared with low-viscosity sodium alginate (4% *w*/*v*), succinic acid (2% *w*/*v*), and CM (0.6% *w*/*v*). After sterilization (121 °C, 15 min), pH was adjusted to 5.6 ± 0.2 with NH_4_OH. Then, to activate calcium alginate cross-linking, a sterile, homogeneous suspension (20 mL) of CaHPO_4_ (0.6% *w*/*v*) was added. The mixture was kept stirring for 30 min at room temperature. Then, the composite of probiotic biomass and CM-Fe_3_O_4_ NPs attached to the cell surface was added to form the encapsulation solution. Finally, the bacterial suspensions with total viable counts between 10^8^ and 10^9^ CFU/mL were kept under constant stirring until spray-drying. For the control solution (M-LGG), the same procedure was carried out, except that the LGG cell culture did not contain CM-Fe_3_O_4_ NPs. Spray-dried powders were collected in sterile glass bottles attached to the bottom of the cyclone and stored at 4 °C until characterization. Encapsulation solutions were prepared in three independent biological replicates, and each formulation was spray-dried in duplicate technical runs.

### 2.6. Evaluation of LGG Survival After Spray Drying Process

#### 2.6.1. Spray Drying Process

For the spray-drying process, a laboratory spray-dryer unit (Büchi B290, Flawil, Switzerland) was used. The process parameters were set as follows: inlet temperature = 130 °C; feed flow rate = 6 mL/min; air flow rate = 45 m^3^/h; outlet temperature = 72–75 °C. These conditions were equal and constant for all experimental trials. Survival assay was performed according to Section 2.9. Spray-drying experiments were performed in two biological replicates, with each replicate analysed in duplicate technical determinations of survival.

#### 2.6.2. Membrane Integrity and LGG Growth Capacity with CM-Fe_3_O_4_ NPs

LGG were cultured in MRS medium, washed with sterile PBS, and stained with SYTO 9 fluorophore, which penetrates only bacteria with intact membranes. After brief incubation in the dark, the samples were mounted on slides and observed by confocal microscopy with excitation at 488 nm, thereby recording the characteristic green fluorescence of viable cells, which allowed assessment of their integrity and viability without interference from the encapsulating matrix or CM-Fe_3_O_4_ NPs.

LGG growth curve was measured. The spray-dried samples were added to sterilized MRS liquid medium, and the bacterial growth was monitored for 24 h. The optical density (OD600) was measured every 2 h using a Genesys 10S (Thermo Scientific, USA). Membrane integrity assays and growth curves were conducted in three biological replicates, with three technical replicates per biological replicate.

### 2.7. Characterization of the Physicochemical Properties of LGG Microcapsules

#### 2.7.1. Morphology of Spray Dried Microcapsules

The micrographs were obtained using a JEOL JSM-6010PLUS/LA (JEOL Ltd., Tokyo, Japan) scanning electron microscope (SEM). The sample was prepared by placing the dry material on an aluminum slide and coating it with a thin layer of gold using sputtering to improve conductivity and prevent surface charge accumulation. The images were acquired with an acceleration voltage of 10 kV, a working distance of 9 mm, and using secondary electron imaging (SEI) mode, which is suitable for observing surface morphology. The magnification used was 1300×, with a reference scale of 10 µm.

#### 2.7.2. Moisture Content

The moisture content of the microcapsules was determined gravimetrically by oven drying at 102 °C until a constant weight was reached. The assay was performed in duplicate.

#### 2.7.3. Determination of Functional Groups Present in Microcapsules by FTIR

The determination of the functional groups present in the structure of microcapsules was performed according to Section 2.3.3.

### 2.8. Evaluation of the Viability of LGG Under In Vitro Gastrointestinal Digestion Conditions

To simulate gastrointestinal tract digestion, the INFOGEST protocol proposed by Brodkorb et al. [27] was used. Briefly, the oral phase was prepared by mixing 1 g of the sample (powder) and 1 mL of simulated salivary fluid (SSF). The final pH of the oral phase was brought to 7.0 and shaken (15 rpm) at 37 °C for 2 min. Next, the gastric phase consisted of adding 4 mL of simulated gastric fluid (SGF) to the oral phase, and the pH was adjusted to 3.0 with the required volume of HCl (1 M). The sample was subjected to agitation (15 rpm) for 2 h at 37 °C. Finally, the intestinal phase was performed by mixing the gastric phase with 8 mL of simulated intestinal fluid (SIF), bile extract (10 mM), and pancreatin (100 U/mL). The final pH of the intestinal phase was adjusted to 7.0 with the required volume of NaOH (1 M) or HCl (1 M), and the mixture was incubated under the same conditions as the gastric phase. After each phase, a viability assay was performed as described in Section 2.9. Simulated digestion assays were performed in three biological replicates, and each biological replicate was plated in triplicate technical replicates for viable counts.

### 2.9. Analytical Methodology

Standard plate colony count determined the survival and viability of LGG after drying. Briefly, the sample (0.1 g) was diluted in sterile 0.1% (*w*/*v*) buffered peptone water in 4.9 mL. Suitable dilutions of the suspension were seeded on MRS agar and incubated 37 °C for 48 h. Logarithmic reduction (LR) was calculated according to standard microbiological practice [28] using the equation:

LR = log_10_(N_0_) − log_10_(N) (1)

where N is the viable count (CFU/g) of the spray-dried powder immediately after drying, and N_0_ is the viable count (CFU/g) of LGG per g of dry matter in the suspension fed to the spray dryer. LR values were calculated for each biological replicate, and the mean ± SD was obtained from three biological replicates × three technical replicates.

### 2.10. Statistical Analysis

Statistical analysis was performed using Design-Expert 6.0 statistical software (Stat-Ease, Minneapolis, MN, USA). All data are expressed as mean ± SD of three biological replicates, each analysed with three technical replicates. ANOVA and Tukey’s test were applied to detect significant differences (*p* < 0.05) (GraphPad Prism 10.6.0).

## 3. Results

### 3.1. Synthesis and Characterization of CM-Fe_3_O_4_ NPs

#### 3.1.1. Optimization of the Size Distribution and Zeta Potential of CM-Fe_3_O_4_ NPs

To evaluate the experimental results, a central composite response surface model was fitted using Design-Expert 6.0 software. This approach allowed the identification of optimal variable levels, the assessment of possible interactions, and the determination of their relevance to the synthesis of iron nanoparticles and in the measurement of zeta potential. The design considered two main factors: the salt concentration ratio FeCl_2_:FeCl_3_ (X_1_) and pH (X_2_), both critical for defining the optimal synthesis conditions. The experimental matrix and the corresponding response variables are summarized in Table 1, where observed values are compared with those predicted by RSM.

The particle size distribution was modeled using a quadratic equation fitted to the experimental data:

(2) $$Y=318.21+51.83X_{1}-61X_{2}+167.97X_{1}^{2}+62.47X_{2}^{2}+175X_{1}X_{2},$$

The zeta potential was modeled using a quadratic equation fitted to the experimental data:

(3) $$Y=-18.78+0.90X_{1}-6.39X_{2}+3.74X_{1}^{2}+3.58X_{2}^{2}+4.42X_{1}X_{2},$$

The ANOVA analysis confirmed that the regression model was statistically significant (*p* < 0.05) (Table 2). The F values obtained for size distribution (15.43) and zeta potential (5.32) indicated a meaningful association between the responses and the evaluated factors. The model showed a strong fit for size distribution (R^2^ = 0.939; adjusted R^2^ = 0.878; Adeq Precision = 10.93) and an acceptable fit for zeta potential (R^2^ = 0.842; adjusted R^2^ = 0.684; Adeq Precision = 7.36), with both Adeq Precision values exceeding the recommended threshold for predictive reliability.

For size distribution, the overall model was significant (*p* = 0.0046), with factor X_2_ exerting a notable effect (*p* = 0.0060). Factor X_1_ did not reach statistical significance (*p* = 0.820) but was retained in the equation to maintain model integrity and avoid bias from variable exclusion. Regarding zeta potential, the model was also significant (*p* = 0.0452), with X_2_ identified as the only factor contributing significantly (*p* = 0.0110).

The lack-of-fit test was significant for both size distribution (*p* = 0.0273) and zeta potential (*p* = 0.0231), suggesting that the models did not fully account for system variability, likely reflecting the inherent complexity of biological responses.

Although some factors showed marginal *p*-values near the significance threshold (*p* ≈ 0.05), these results were considered biologically relevant given the inherent variability in chia mucilage composition. Natural biopolymers often present batch-to-batch differences in polysaccharide content and hydration capacity, which can influence nanoparticle size distribution and zeta potential. Therefore, the observed lack of fit likely reflects biological variability rather than methodological error, supporting the robustness of the synthesis approach under realistic conditions.

**Figure 1:**
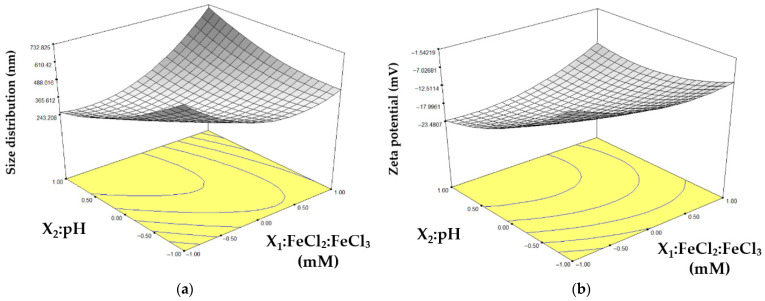
3D response surface plots, (**a**) size distribution, (**b**) zeta potential of CM-Fe_3_O_4_ NPs.

By applying the predictive models, the experimental conditions that minimized both particle size distribution and zeta potential were identified. The optimal parameters corresponded to a pH of 11 and salt concentrations of 40/20 mM, yielding nanoparticles with an average diameter of 265 nm and a zeta potential of −23.65 mV (Figure 1). Replication of these conditions confirmed the model’s accuracy, producing particles of 226 nm with a zeta potential of −24.55 mV.

#### 3.1.2. Characterization of CM-Fe_3_O_4_ NPs

**Figure 2:**
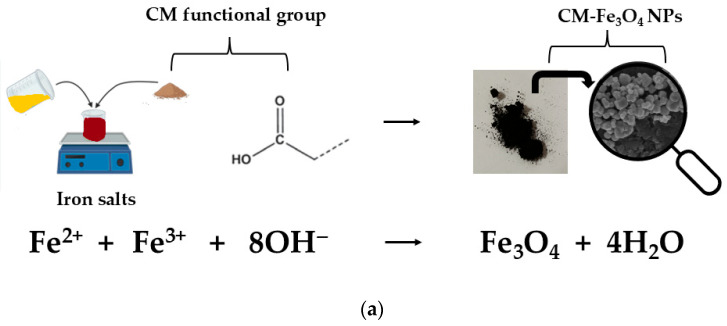

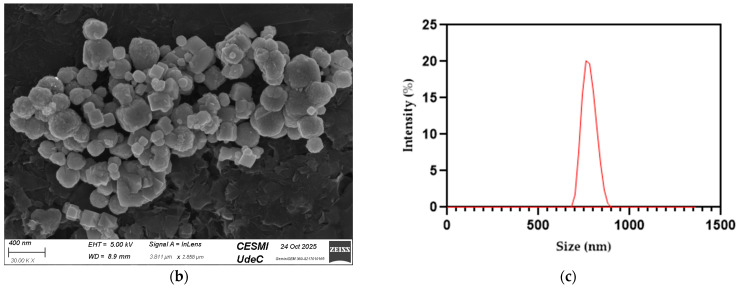
Synthesis and characterization of CM-Fe_3_O_4_ NPs: (**a**) schematic molecular diagram showing hydroxyl groups of chia mucilage interacting with Fe^2+^/Fe^3+^ ions; (**b**) morphology observed by FE-SEM (GeminiSEM 360, ZEISS, Germany, 5.0 kV, 30,000× magnification, scale bar 400 nm); (**c**) hydrodynamic size distribution measured by DLS (Zetasizer Nano ZS90, Malvern Instruments, UK, 1 mg/mL suspension in distilled water).

The biosynthesis of CM-Fe_3_O_4_ NPs was achieved by combining CM extract with iron salts, where hydroxyl groups (–OH) acted as ligands, forming complexes with the metal ions (Figure 2a). Adjustment of pH with NaOH resulted in a progressive color change to deep black–brown, and after drying, a black powder was obtained, confirming the formation of CM-Fe_3_O_4_ NPs [16].

FE-SEM imaging revealed spherical particles with mucilage coating, showing sizes between 50–300 nm (Figure 2b). In contrast, DLS analysis showed a larger hydrodynamic size between 600–900 nm (Figure 2c), attributable to coating hydration and slight aggregation in suspension [16]. These differences highlight the hybrid nature of the system: a small and stable inorganic core surrounded by an amorphous organic matrix, which increases the apparent size without necessarily implying greater cytotoxicity [29].

**Figure 3:**
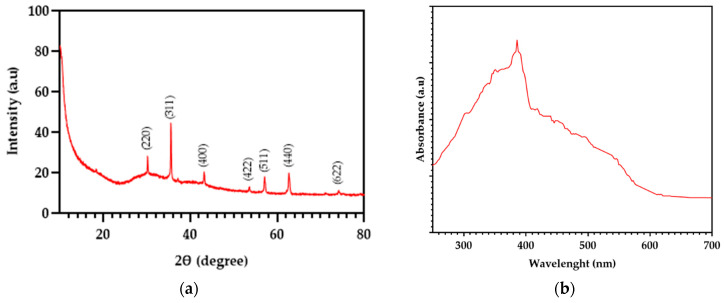

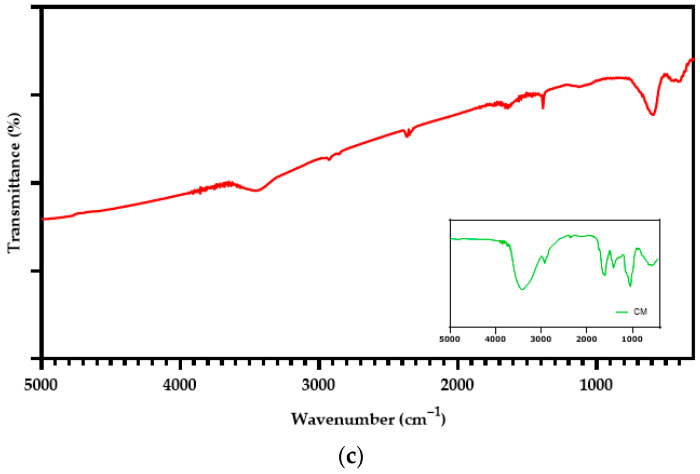
Characterization of CM-Fe_3_O_4_ NPs: (**a**) XRD pattern obtained with Siemens D5000 (Munich, Germany), showing magnetite crystalline peaks at 2θ = 30°, 36°, and 63°; (**b**) UV–Vis absorption spectrum recorded with Genesys 10S spectrophotometer (Thermo Scientific, USA), highlighting bands between 375–435 nm; (**c**) FT–IR spectra measured with Bruker Vector 22 (Germany), showing hydroxyl (–OH), ester (C=O), and Fe–O stretching vibrations.

XRD patterns confirmed magnetite (Fe_3_O_4_) as the main crystalline phase (JCPDS 65-3107), demonstrating that the Fe_3_O_4_ structure remained stable during synthesis with CM –CM-Fe_3_O_4_ NPs– (Figure 3a). The broad diffraction peaks at 2θ values of 30°, 36°, and 63° (Figure 3a), corresponding to the (220), (311), and (440) planes, suggest the presence of small crystallite domains [16]. UV–Vis spectroscopy (Figure 3b) revealed a strong absorption band with inflections between 375–435 nm, consistent with the optical response of magnetite (Fe_3_O_4_) [30]. FT–IR spectra (Figure 3c) displayed broad peaks at 3350–3500 cm^−1^, attributed to hydroxyl (–OH) groups, indicative of hydrogen bonding and surface functionalities [31]. Additional ester (C=O) bands were observed, suggesting protein incorporation from the CM extract [31]. Characteristic Fe–O stretching vibrations appeared at 410 cm^−1^ (tetrahedral coordination) and 610 cm^−1^ (octahedral coordination), confirming the formation of iron oxide with a spinel structure [16].

Compared to other natural stabilizers such as pectin, alginate, or chitosan [10,11,12,13], chia seed mucilage provided distinctive advantages in the biosynthesis of CM-Fe_3_O_4_ NPs, including strong water-binding capacity and food-grade biocompatibility. This highlights the potential of CM as a sustainable alternative to conventional coatings.

### 3.2. Preparation of the Bacterial Culture with CM-Fe_3_O_4_ NPs

**Figure 4:**
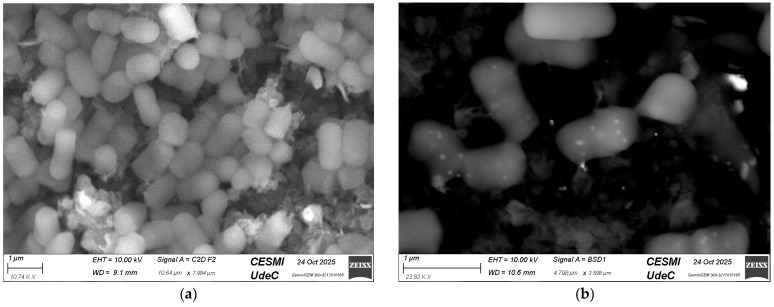
SEM micrographs of LGG cultures: (**a**) control cells without nanoparticles; (**b**) LGG with CM-Fe_3_O_4_ NPs attached to the cell surface (JEOL JSM-6010PLUS/LA (JEOL Ltd., Tokyo, Japan), 10 kV, 1300× magnification, scale bar 10 µm). White dots correspond to nanoparticles immobilized on bacterial membranes.

SEM analysis showed clear morphological differences between LGG cultures with and without CM-Fe_3_O_4_ NPs. In Figure 4b, the white dots indicate nanoparticles attached to bacterial cell surfaces, demonstrating the successful interaction between the nanostructures and the microorganisms. In contrast, the control sample without CM-Fe_3_O_4_ NPs displayed a more uniform distribution of rod-shaped LGG cells, with smoother surfaces and no signs of nanoparticle incorporation. These results suggest that CM-Fe_3_O_4_ NPs affect the microstructural organization of the system, potentially enhancing the stability and functionality of probiotics [14,16].

### 3.3. Evaluation of the Survival of the LGG Co-Encapsulated with CM-Fe_3_O_4_ by Spray Drying

#### 3.3.1. Survival of M-LGG-CM-Fe_3_O_4_ by Spray Drying

Table 3 shows the viability of LGG after spray drying for both the control microcapsules (M-LGG) and the co-encapsulation system containing iron oxide nanoparticles synthesised with CM (M-LGG-CM-Fe_3_O_4_). High cell viability was maintained in both formulations, and no statistically significant differences (*p* < 0.05) were observed between treatments. These results indicate that the incorporation of nanoparticles did not compromise bacterial survival during the spray drying.

Moisture content analysis also revealed no significant differences (*p* < 0.05) between the two types of microparticles. The values obtained are within the optimal range for microbial and chemical stability, supporting the extended shelf life of LGG powders [32].

**Figure 5:**
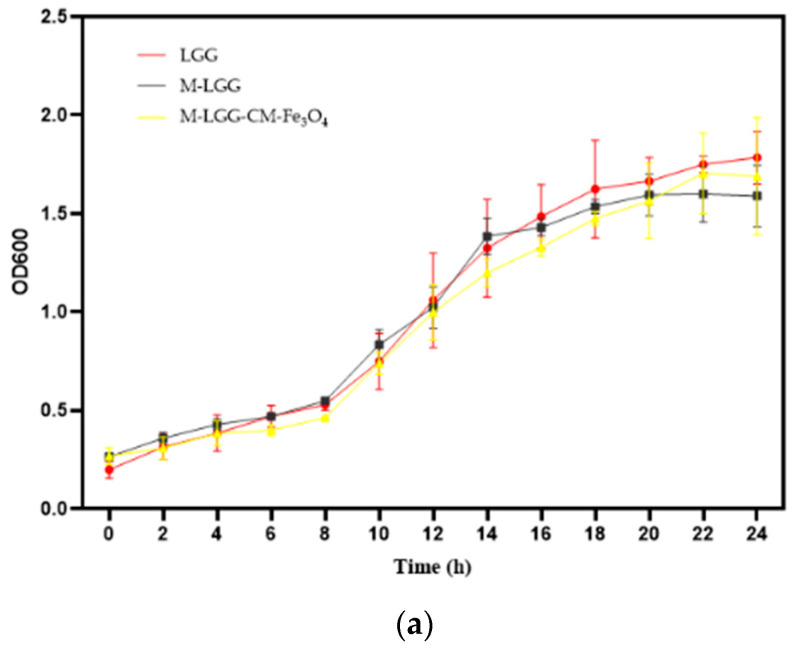

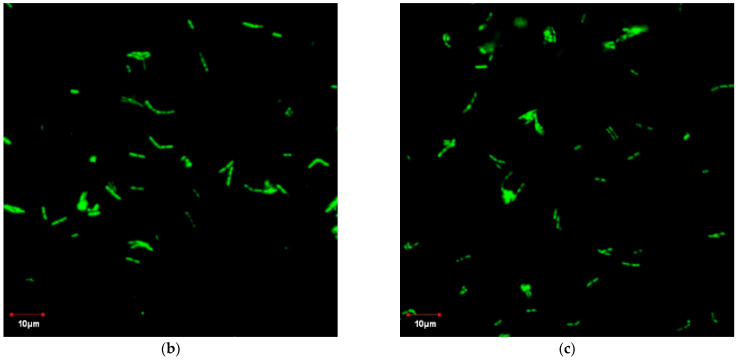
Viability of LGG, (**a**) LGG growth curves, (**b**) M-LGG: confocal image of the control microcapsule, (**c**) M-LGG-CM-Fe_3_O_4_: confocal image of the microcapsule with CM-Fe_3_O_4_ NPs. Means ± standard deviation (*n* = 3).

As shown in Figure 5a, both spray-dried probiotic bacteria exhibited typical bacterial growth, characterized by lag, exponential, and stationary phases. Growth then matched that of free LGG, with no signs of delayed bacterial growth after encapsulation.

Confocal micrographs (Figure 5b,c) revealed a predominance of viable LGG cells. The consistently high viability observed in both images demonstrates that the experimental conditions effectively maintained bacterial integrity after spray drying, in both the control sample (Figure 5a) and the co-encapsulation system with CM-Fe_3_O_4_ NPs (Figure 5b). The bacterial cells appeared as bright green rod-shaped structures, with no significant morphological differences between treatments. This result is predictable due to the lack of intrinsic fluorescence of the CM-Fe_3_O_4_ NPs.

#### 3.3.2. Characterization of M-LGG-CM-Fe_3_O_4_ NPs

**Figure 6:**
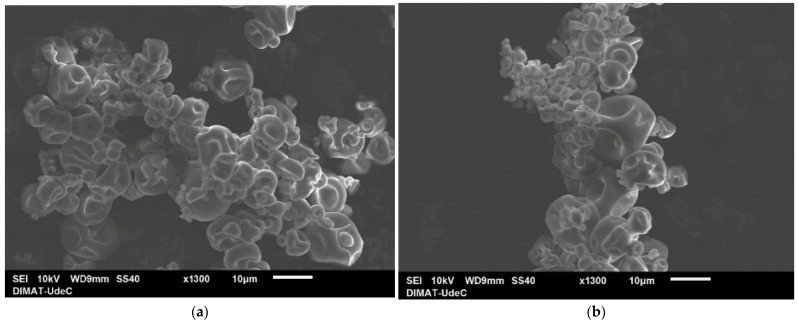
SEM micrographs of LGG microcapsules, (**a**) M-LGG: SEM image of the control microcapsule, (**b**) M-LGG-CM-Fe_3_O_4_: SEM image of the microcapsule with CM-Fe_3_O_4_ NPs.

Both SEM micrographs confirm effective encapsulation of LGG using alginate and CM. However, the inclusion of CM-Fe_3_O_4_ NPs (Figure 6b) appears to have resulted in a more compact structure, with a more defined morphology and a clear presence of inorganic elements, possibly enhancing the structural and functional stability of the encapsulation [16].

**Figure 7:**
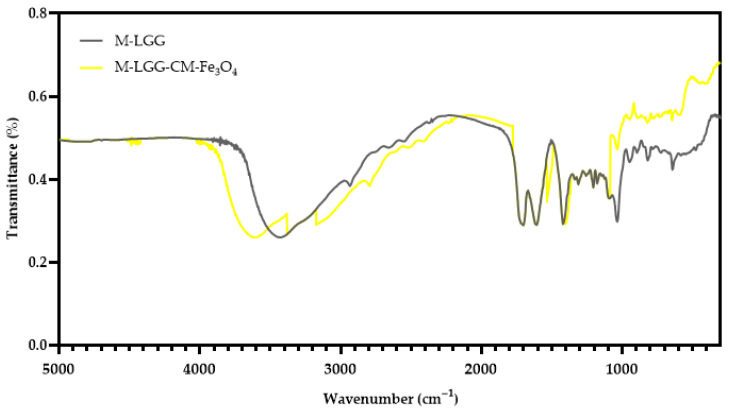
Functional group analysis using FTIR. M-LGG: control microcapsule. M-LGG-CM-Fe_3_O_4_: microcapsule with CM-Fe_3_O_4_ NPs.

Figure 7 shows the FTIR spectra of M-LGG and M-LGG-CM-Fe_3_O_4_. The band at 1740 cm^−1^ can be attributed to the vibrations of organic acids. The peak observed near 1600 cm^−1^ corresponds to the amide I band for C=O stretching and indicates the presence of protein portions in galactomannan [5]. The bands at around 1400 cm^−1^ were ascribed to the CH_2_ vibrational modes, and the group of bands in the 1200–850 cm^−1^ region, in bands attributed to the glycosidic linkage of COH [33]. The band characterized the spectrum of CM-Fe_3_O_4_ (M-LGG-CM-Fe_3_O_4_) at 550 cm^−1^, ascribed to Fe–O vibrations, and the shoulder at 620 cm^−1^ corresponded to Fe-O-O vibrations [16,34]. The band at 1620 cm^−1^ indicates the presence of humidity in the after-spray drying process [8].

### 3.4. Evaluation of the Viability of LGG Under Gastrointestinal Digestion Conditions

**Figure 8:**
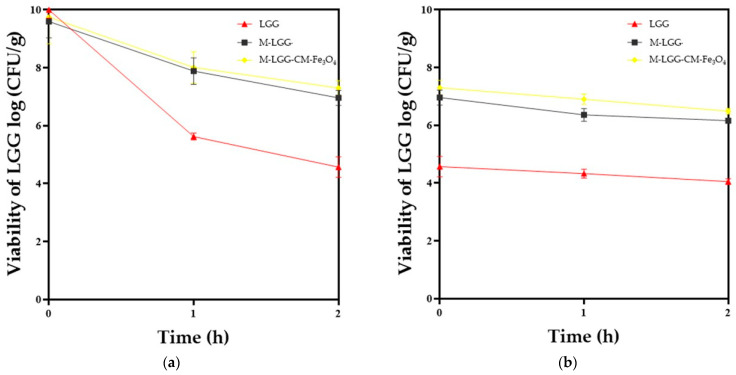
Viability of gastrointestinal digestion, (**a**) bacterial counts of LGG, M-LGG, and M-LGG-CM-Fe_3_O_4_ after exposure to simulated gastric fluid, (**b**) bacterial counts of LGG, M-LGG, and M-LGG-CM-Fe_3_O_4_ after exposure to simulated intestinal fluid. Means ± standard deviation (*n* = 3).

The release and viability of LGG were evaluated through in vitro gastrointestinal digestion. Although the gastric phase was the primary focus, both microcapsule types were exposed to SGF and SIF, and their performance was compared with that of free LGG cells (control) (Figure 8). In SGF, after 2 h of exposure, the microcapsules containing LGG co-encapsulated with CM-Fe_3_O_4_ exhibited slightly higher viability than M-LGG; however, the difference was not statistically significant (*p* < 0.05). These findings suggest that encapsulating agents such as CM provide protective effects under gastric conditions, thereby facilitating the controlled release of LGG [35]. Verification of this behavior in the intestinal phase was therefore necessary. During the transition to SIF, LGG was progressively released until reaching the intestinal environment, where conditions (pH 7.0–8.0) favor complete release [29]. After 2 h of exposure in SIF, viability analysis revealed that M-LGG-CM-Fe_3_O_4_ maintained higher viability (7.23 log CFU/g) during the first hour compared with both the control microcapsule and free LGG cells. This outcome indicates that co-encapsulation enhanced resistance to the adverse conditions of the preceding gastric phase, including low pH (≈3) and enzymatic activity. Under in vitro gastrointestinal conditions, co-encapsulation of LGG with CM-Fe_3_O_4_ improved survival in the gastric phase and significantly enhanced viability in the initial intestinal phase. These findings confirm the protective effect of the nanocomposite system and demonstrate its effectiveness in safeguarding LGG against damaging digestive conditions.

## 4. Discussion

The present study demonstrated that chia mucilage can act as a natural stabilizer for the synthesis of CM-Fe_3_O_4_ NPs. The functional groups of CM, particularly hydroxyl, carbonyl, and carboxyl moieties, contributed to nanoparticle stabilization, as confirmed by FTIR analysis [26]. At the molecular level, hydroxyl groups can form hydrogen bonds with the surface of Fe_3_O_4_, while carbonyl and carboxyl groups can coordinate with Fe^2+^/Fe^3+^ ions, thereby reducing aggregation and promoting colloidal stability [27]. This dual interaction explains the hydrated coatings observed in DLS analysis, which are consistent with biopolymer-based nanostructures reported in other systems [15,28]. The presence of mucilage polysaccharides also provides steric hindrance, preventing particle agglomeration and ensuring dispersion stability [29]. These findings suggest that chia mucilage provides a sustainable alternative to synthetic stabilizers, aligning with current trends in bio-based nanomaterials [30].

Encapsulation of *Lactobacillus rhamnosus* GG with CM-Fe_3_O_4_ NPs did not compromise bacterial viability during spray drying. Survival values expressed as logarithmic reduction (LR) remained below 1 log_10_, indicating minimal loss compared to the initial suspension. This performance is comparable to that of other encapsulation systems using polysaccharides [31], but the addition of CM-Fe_3_O_4_ NPs enhanced the structural integrity of the microcapsules. At the molecular level, the mucilage matrix likely formed a protective hydrocolloid network around LGG cells, while Fe_3_O_4_ nanoparticles contributed to cross-linking interactions that reduced mechanical stress during atomization [30,32]. The iron oxide surface may also interact electrostatically with negatively charged bacterial cell walls, stabilizing their position within the matrix [30,33]. Spray drying was selected over freeze-drying or extrusion because it allows rapid water removal, low residual moisture, and scalability for industrial applications [34]. In contrast, freeze-drying often results in porous structures with higher oxygen permeability, while extrusion can lead to uneven particle distribution. The improved survival highlights the potential of combining biopolymers with magnetic nanoparticles to strengthen probiotic delivery systems [16,35].

During the gastrointestinal digestion simulation, the co-encapsulated LGG showed significantly higher viability rates than the controls. In the gastric phase, the log reduction (LR) was lower for nanoparticle-containing formulations, whereas in the intestinal phase, viability remained above 7 log_10_ CFU/g. These results indicate that CM-Fe_3_O_4_ NPs modulate matrix degradation and promote controlled release, thereby improving probiotic resilience under harsh conditions. Mechanistically, the mucilage–nanoparticle network likely slowed proton diffusion and enzyme penetration, delaying capsule disintegration [30,31]. The hydrated mucilage chains may have acted as a diffusion barrier, while Fe_3_O_4_ nanoparticles contributed to the mechanical reinforcement of the capsule wall [36,37]. Such findings are consistent with previous studies reporting that nanoparticle incorporation can enhance protection against acidic and enzymatic stress [14,15]. Furthermore, the presence of Fe_3_O_4_ may have contributed to electrostatic interactions with digestive enzymes, reducing their accessibility to the encapsulated bacteria [18,38,39]. This mechanism is supported by reports that iron oxide nanoparticles can adsorb proteins and enzymes, altering their activity [40].

The use of LR as a metric, rather than percentage viability, provides a more standardized and comparable measure of probiotic survival under stress conditions [41,42].

The statistical analysis provided insights into the influence of pH and FeCl_3_/FeCl_2_ ratio on nanoparticle size and zeta potential. The response surface model captured the main trends but did not fully account for all variability, as the residuals indicated deviations from the predicted values. This limitation suggests that additional factors, such as ionic strength, mucilage composition, or drying kinetics, may also contribute to nanoparticle behavior. Therefore, statistical adjustment should be interpreted as indicative rather than exhaustive, and future studies should incorporate broader experimental conditions to refine model accuracy [43]. Importantly, while ANOVA confirmed significant effects, the model residuals highlight the complexity of biopolymer–nanoparticle systems, which cannot be fully captured by simplified statistical approaches. This is consistent with previous reports, where biopolymer heterogeneity introduced variability not accounted for by linear models [44].

From an application perspective, integrating CM-Fe_3_O_4_ NPs into probiotic encapsulation systems is a promising approach to enhancing microbial survival and functionality. The combination of natural polysaccharides and magnetic nanoparticles offers dual benefits: biocompatibility and mechanical reinforcement. However, the findings should be considered preliminary, and further research is needed to optimize nanoparticle synthesis, evaluate long-term stability, and validate performance in food matrices. Future studies should also explore the scalability of this approach, the sensory impact on food products, and the regulatory implications of incorporating magnetic nanoparticles into edible systems.

To fully exploit the potential of CM-Fe_3_O_4_ NPs in probiotic encapsulation and functional food applications, several aspects warrant further investigation. First, controlled iron release from the nanoparticles should be evaluated under gastrointestinal conditions to determine whether CM-Fe_3_O_4_ can act as a safe and effective iron supplement. Such studies would clarify whether the system can simultaneously deliver probiotics and contribute to dietary iron intake, addressing nutritional deficiencies in vulnerable populations. Second, cytotoxicity assays using relevant cell lines (e.g., intestinal epithelial cells, macrophages) are essential to confirm biocompatibility and rule out adverse effects associated with nanoparticle exposure [18,29,41]. These assays would provide mechanistic insights into nanoparticle–cell interactions, including potential oxidative stress or inflammatory responses. Finally, in vivo experiments are necessary to validate the protective effects observed in vitro and to explore the potential of magnetically guided delivery for targeted probiotic release [42]. Magnetic guidance could open new avenues for site-specific probiotic administration, enhancing colonization efficiency and therapeutic outcomes. By addressing these issues, the proposed system could contribute to the development of next-generation functional foods with improved probiotic delivery and iron release systems.

## 5. Conclusions

In this study, we demonstrated the biosynthesis of Fe_3_O_4_ nanoparticles using chia seed mucilage and their application for the co-encapsulation of *Lactobacillus rhamnosus* GG via spray drying. The nanoparticles exhibited favorable physicochemical properties (particle size, zeta potential, morphology) that contributed to improved probiotic survival under simulated gastrointestinal conditions, particularly during the gastric and initial intestinal phases. At the molecular level, the interactions between mucilage functional groups and Fe^2+^/Fe^3+^ ions provided colloidal stability, while the hydrocolloid network reinforced capsule integrity, supporting the dual role of CM-Fe_3_O_4_ in probiotic protection and potential iron supplementation.

These findings highlight the multifunctionality of the CM-Fe_3_O_4_ system: stabilization of probiotics, modulation of gastrointestinal release, and potential contribution to dietary iron intake. Such attributes support its relevance for next-generation functional foods. Nevertheless, limitations such as variability in mucilage composition, incomplete statistical adjustment of the response surface model, and uncertainties regarding long-term storage stability should be addressed in future work. Cytotoxicity assays and mechanistic studies will also be necessary to confirm biocompatibility and rule out adverse effects associated with nanoparticle exposure.

Further studies should explore the incorporation of this nanocomposite into complex food matrices, validate its performance under industrial conditions, and assess its efficacy in vivo. Controlled iron release under gastrointestinal conditions should be evaluated to determine whether CM-Fe_3_O_4_ can act as a safe and effective iron supplement. In vivo experiments will be essential to validate the protective effects observed in vitro and to explore the potential of magnetically guided delivery for targeted probiotic release. Overall, this research fills an important gap by integrating mucilage-mediated nanoparticle synthesis with probiotic encapsulation under physiologically relevant conditions, while opening new perspectives for multifunctional applications in food biotechnology.

## Figures and Tables

**Table 1:** Design matrix of experiments with three central points in relation to size distribution and zeta potential of CM-Fe_3_O_4_ NPs.

Experiment	X_1_: FeCl_3_:FeCl_2_ (mM/mM)	X_2_: pH	Y: Size Distribution ^†^ (nm) (Actual)	Y: Size Distribution (nm) (Predicted)	Y: Z Potential ^†^ (mV) (Actual)	Y: Z Potential (mV) (Predicted)
1	40/20	9	740.00 ± 0.88	732.82	−4.24 ± 0.62	−1.54
2	60/30	10	343.00 ± 1.29	318.21	−21.01 ± 0.53	−18.78
3	80/40	10	458.00 ± 0.62	538.02	−11.06 ± 0.72	−14.14
4	80/40	11	753.00 ± 1.33	714.49	−13.01 ± 0.32	−12.54
5	40/20	10	423.00 ± 1.42	434.35	−12.75 ± 0.22	−15.93
6	60/30	10	340.00 ± 1.08	318.21	−20.05 ± 0.21	−18.78
7	80/40	9	528.00 ± 0.78	486.49	−11.25 ± 0.33	−8.58
8	40/20	11	265.00 ± 1.42	260.82	−23.65 ± 0.46	−23.17
9	60/30	11	277.00 ± 1.25	319.68	−20.65 ± 0.51	−21.60
10	60/30	10	363.00 ± 1.28	318.21	−21.59 ± 0.29	−18.78
11	60/30	9	393.00 ± 1.37	318.21	−3.43 ± 0.31	−8.80

^†^ Mean values of two replicates, except in the central point where there were three replicates.

**Table 2:** Analysis of variance (ANOVA) of the fitted quadratic model for size distribution and zeta potential of CM-Fe_3_O_4_ NPs.

Source	Sum of Squares	DF	Mean Square	*F* Value	*p* Value
Size distribution					
Model	263,804.94	5	52,760.99	15.43	0.0046
$X_{1}$	16,120.17	1	16,120.17	4.72	0.0820
$X_{2}$	22,326.00	1	22,326.00	6.53	0.0509
$X_{1}^{2}$	71,478.40	1	71,478.40	20.91	0.0060
$X_{2}^{2}$	9887.50	1	9887.50	2.89	0.1498
$X_{1}X_{2}$	122,500.00	1	122,500.00	35.83	0.0019
Residual	17,093.96	5	3418.79		
Lack of Fit	16,781.30	3	5593.77	35.78	0.0273
Pure Error	312.67	2	156.33		
Total	280,898.91	10			
Zeta potential					
Model	420.94	5	84.19	5.32	0.0452
$X_{1}$	4.84	1	4.84	0.31	0.6040
$X_{2}$	245.50	1	245.50	15.51	0.0110
$X_{1}^{2}$	35.50	1	35.50	2.24	0.1944
$X_{2}^{2}$	32.44	1	32.44	2.05	0.2116
$X_{1}X_{2}$	77.97	1	77.97	4.93	0.0772
Residual	79.12	5	15.82		
Lack of Fit	77.90	3	25.97	42.51	0.0231
Pure Error	1.22	2	0.61		
Total	500.06	10			

**Table 3:** Survival and moisture of M-LGG-CM-Fe_3_O_4_ after encapsulation by spray-drying at 130 °C, expressed as logarithmic reductions.

Microcapsule	Before Drying log N_0_ (CFU/g)	After Drying log N (CFU/g)	log Reduction (log N_0_ − log N)	Moisture (%)
M-LGG	10.74 ± 0.17	10.71 ± 0.45	0.03 ± 0.03 ^a^	9.11 ± 0.18 ^a^
M-LGG-CM-Fe_3_O_4_	10.35 ± 0.24	10.31 ± 0.43	0.04 ± 0.03 ^a^	8.98 ± 0.34 ^a^

Means ± standard deviation (*n* = 3), with equal superscript letters in the same line indicating no significant differences (*p* < 0.05) between the microcapsules of LGG. log N is the LGG concentration per g of the spray-dried powder, and log N_0_ is the LGG concentration per g of dry matter in the suspension fed to the dryer.

## Data Availability

The original contributions presented in the study are included in the article, further inquiries can be directed to the corresponding author.

## Abbreviations

CM	Chia Seed Mucilage
CLAMs	Cross-Linked Alginate Matrices
CM Fe_3_O_4_	Chia mucilage mediated magnetite nanoparticles
LGG	*Lactobacillus rhamnosus* GG
NPs	Nanoparticles
M-LGG	Microencapsulated *Lactobacillus rhamnosus* GG
M-LGG-CM Fe_3_O_4_	Co encapsulation of LGG with CM Fe_3_O_4_ nanoparticles
SEM	Scanning Electron Microscopy
FE-SEM	Field Emission Scanning Electron Microscopy
XRD	X ray Diffraction
UV-vis	Ultraviolet–Visible Spectroscopy
FT-IR	Fourier Transform Infrared Spectroscopy
ANOVA	Analysis of Variance
GI	Gastrointestinal
CFUs	Colony Forming Units
SD	Spray Drying
kV	Kilovolt
